# Ionic Channels as Potential Therapeutic Targets for Erectile Dysfunction: A Review

**DOI:** 10.3389/fphar.2020.01120

**Published:** 2020-07-24

**Authors:** Anderson Fellyp Avelino Diniz, Rafael Carlos Ferreira, Iara Leão Luna de Souza, Bagnólia Araújo da Silva

**Affiliations:** ^1^ Programa de Pós-Graduação em Produtos Naturais e Sintéticos Bioativos, Universidade Federal da Paraíba, João Pessoa, Brazil; ^2^ Departamento de Ciências Biológicas e da Saúde, Universidade Estadual de Roraima, Boa Vista, Brazil

**Keywords:** canalopathies, erectile dysfunction, Ca^2+^-activated K^+^ channels, KCNQ-encoded voltage-dependent K^+^ channels, transient receptor potential channels, calcium-activated chloride channels

## Abstract

Erectile dysfunction (ED) is a prevalent condition, especially in men over 40 years old, characterized by the inability to obtain and/or maintain penile erection sufficient for satisfactory sexual intercourse. Several psychological and/or organic factors are involved in the etiopathogenesis of ED. In this context, we gathered evidence of the involvement of Large-conductance, Ca^2+^-activated K^+^ channels (BK_Ca_), Small-conductance, Ca^2+^-activated K^+^ channels (SK_Ca_), KCNQ-encoded voltage-dependent K^+^ channels (K_V_7), Transient Receptor Potential channels (TRP), and Calcium-activated Chloride channels (CaCC) dysfunctions on ED. In addition, the use of modulating agents of these channels are involved in relaxation of the cavernous smooth muscle cell and, consequent penile erection, suggesting that these channels are promising therapeutic targets for the treatment of erectile dysfunction.

## Introduction

Erectile dysfunction (ED) is a persistent inability to achieve and/or maintain a penile erection enough for satisfactory sexual intercourse (McCabe et al., 2016). Predominantly a vascular disorder, ED affects both physical and psychological health, having a direct impact on men**’**s life quality and their sexual partners, mainly due to a reduction in self-esteem and impairment of interpersonal relationships (Boddi et al., 2015; Nguyen et al., 2017; Burnett et al., 2018). It mainly affects men after 40 years of age and it has estimated that over 150 million worldwide have some degree of this dysfunction (Ayta et al., 1999; Grover et al., 2006; Riedner et al., 2011; Tabosa et al., 2017). However, even if the increasing in cases with aging is evident, ED is not an inevitable consequence of aging, which makes it possible to increase the estimate of the average world prevalence (Seftel, 2011; Shamloul and Ghanem, 2013; Yafi et al., 2016; Gabrielson et al., 2019).

Psychological and organic factors such as anxiety, stress, depression, or vascular and hormonal dysfunctions may cause imbalance of the contractile and relaxing mechanisms of the cavernous smooth muscle culminating in the development of ED (Andersson, 2001; Fumaz et al., 2017; Mitidieri et al., 2020).

In recent years, researching involving the flaccidity and penile erection has focused mainly on molecular mechanisms. In this sense, several neurotransmitters, second messengers, reactive oxygen species (ROS), growth factors, hormones, and ion channels have been characterized as important components of the complex erection process, leading to the discovery of new therapeutic targets for the treatment of ED. The search for new therapeutic alternatives for erectile dysfunction is associated with refractoriness to conventional treatments observed in part of the male population. Given this, this review will focus on providing an update on the importance of some ion channels involved in the regulation of intracellular signaling and tone of cavernous smooth muscle and their potential as therapeutic targets to the development of new drugs to treatment of erectile dysfunction.

## Etiological Factors of Erectile Dysfunction

Multifactorial nature of ED is evident and, population studies have shown that several conditions involving vascular abnormalities such as hypertension, aging, physical inactivity, dyslipidemia, diabetes, insulin resistance, and obesity are among the major risk factors that favor the development of ED in man and animal models (Musicki et al., 2010; Kaya et al., 2015; Maseroli et al., 2015). In addition, studies have shown that ED is a predictive factor for the development of cardiovascular disease and may be a potent marker for screening for silent coronary disease (Phe and Roupret, 2012; Gandaglia et al., 2016; Capogrosso et al., 2019; Orimoloye et al., 2019).

The causes of ED are directly related to biopsychosocial processes that involve psychological, endocrine, vascular, and neurological coordination (Prieto, 2008), and can be classified etiologically as psychological, organic or mixed, where there is a combination of both factors (Ayta et al., 1999; Riedner et al., 2011; Yafi et al., 2016).

The most common psychogenic factors include performance anxiety, psychiatric disorders such as anxiety, stress and depression, and relationship conflicts that culminate in reduced sexual libido or fear of failure during intercourse. Organic factors include neurological, endocrine and vascular causes (Fauci et al., 2012; Swerdloff and Wang, 2012; Mccabe and Althof, 2014). Neurological or neurogenic ED have been represented, mainly, by signaling deficiency through penile innervations (Brackett et al., 2010). Neurological causes have been responsible for approximately 10 to 19% of ED cases and are among those causes, such as Parkinson**’**s disease, dementia, demyelinating disease and spinal cord injury at levels affecting erection and/or ejaculation (Keller et al., 2012; Ludwig and Phillips, 2014; Antuña et al., 2015). Reduced testosterone levels, hormone responsible for increasing endothelial nitric oxide synthase (eNOS) expression, and reduced protein expression of Small G protein GTP-binding/Rho-associated protein kinase (RhoA/ROCK) pathway characterize endocrine ED (Lugg et al., 1996; Mills et al., 2001; Hu et al., 2009; Sopko et al., 2014). In addition, the main endocrine causes are diabetes *mellitus*, metabolic syndrome (MS) and changes in sex hormones (Özdemirci et al., 2001; Swerdloff and Wang, 2012; Ludwig and Phillips, 2014; Papagiannopoulos et al., 2015). Arterial traumatic disease, atherosclerosis and systemic arterial hypertension represent the main causes of vascular etiology (Perticone et al., 2005; Fauci et al., 2012), and are directly related to endothelial dysfunction, which may result from imbalance of NO, increased sympathetic activity and structural changes that reduce the relaxing capacity of the corpus cavernosum of the penis (Corona et al., 2006; Jackson, 2007; Swerdloff and Wang, 2012).

Moreover, aging is the major risk factor for ED and both the prevalence and severity of the disease increase with aging, so it is usually caused by the presence of neural and endothelial dysfunction (El-Sacca, 2007; Lewis et al., 2012).

## Physiological Mechanisms of Flaccidity and Penile Erection

Penis is the male genital and copulatory organ responsible for the elimination of urine and sexual intercourse (Sachs and Meisel, 1988; Katz, 2002). It can be divided into three parts: base, body and glans. Penis base is formed by three cylindrical structures corresponding to two corpus cavernosum and a corpus spongiosum (Eardley et al., 2013).

Corpus cavernosum comprise two parallel smooth muscle cylindrical structures surrounded by a compact fibrous tissue structure, known as the albuginia tunic, which gives the penis rigidity, strength and flexibility (Awad et al., 2011; Doyle, 2011; Avery and Scheinfeld, 2015).

Smooth muscle of the corpus cavernosum is important for erection and maintenance of penile flaccidity. Most of the time, smooth muscle cells remain in their contracted state, which limits the filling of vessels that nourish the corpus cavernosum with blood and, consequently, favor the maintenance of flaccidity (Thomas and Bohnen, 2005; Andersson, 2011). On the other hand, due to neurovascular modulation mediated by psychological and hormonal factors, cavernous smooth muscle cells, in a coordinated manner, may be in their relaxed state, from a complex interaction between the central nervous system (CNS) and local stimuli. As a result, the filling of the corpus cavernosum with increasing intracavernous pressure promote penile erection. Thus, muscle cells of the corpus cavernosum operate together in synchronicity, as they not only contract spontaneously in a coordinated manner, but also relax synchronously (Brownstein et al., 2017).

Penile flaccidity process primarily have been stimulated by the sympathetic nervous system, where the release of norepinephrine (NA) by adrenergic neurons stimulates its α_1_ and α_2_ receptors in the smooth muscle of the penile vessels and corpus cavernosum, inducing its contraction and reduction of the blood flow (Goldstein, 2000; Gur et al., 2012; Traish et al., 2015).

**Figure 1:**
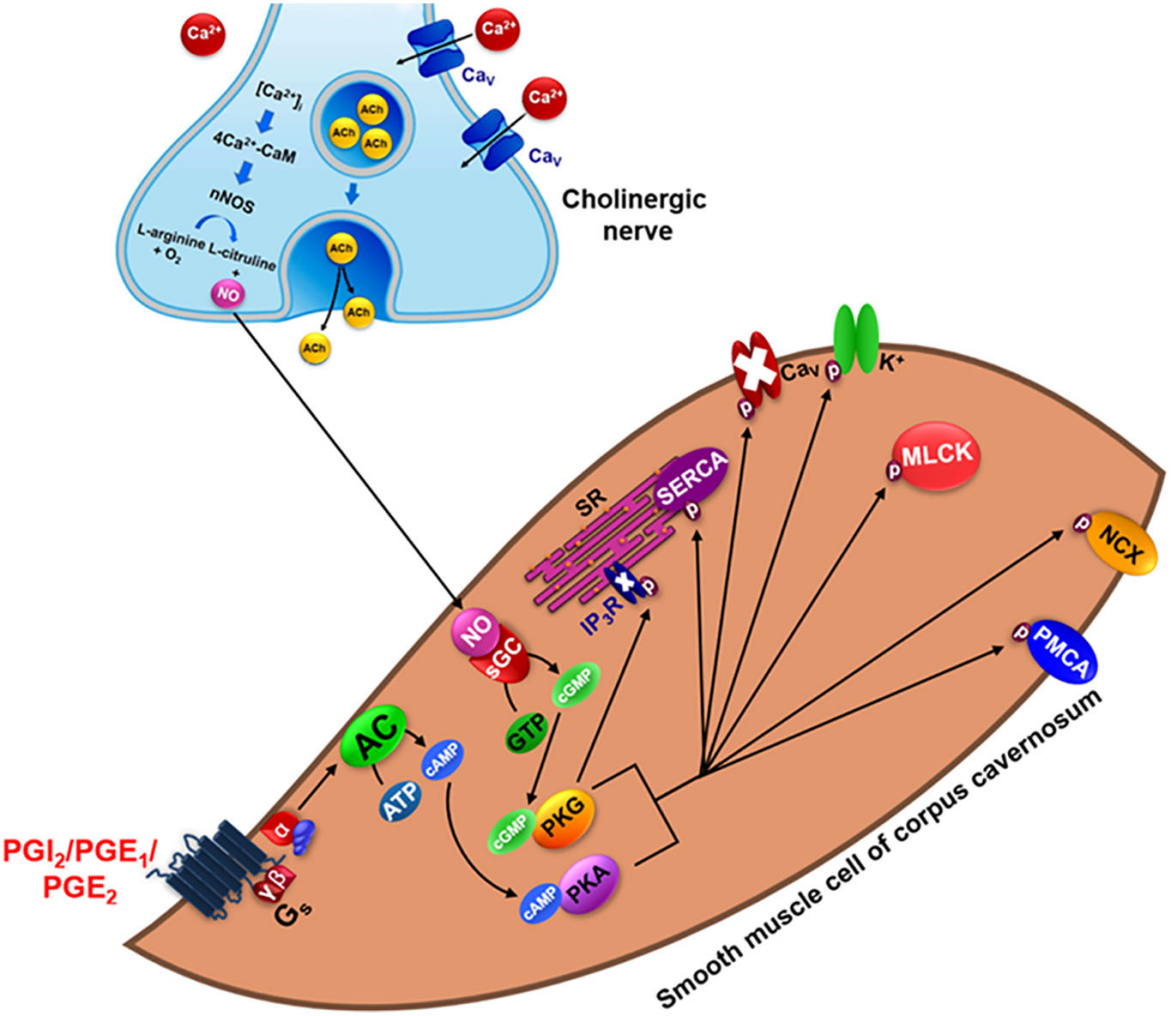
Physiological mechanism of relaxation in the cavernous smooth muscle. CaM, calmodulin; nNOS, neuronal nitric oxide synthase; NO, nitric oxide; Ca_V_, voltage-dependent Ca^2 +^ channels; PGI_2_, prostacyclin; PGE_1/2_, prostaglandin E type 1 and 2; AC, adenylyl cyclase; ATP, adenosine triphosphate; cAMP, cyclic adenosine monophosphate; PKA, cAMP dependent protein kinase; sGC, soluble guanylyl cyclase; GTP, guanosine triphosphate; cGMP, cyclic guanosine monophosphate; PKG, cGMP-dependent protein kinase; GMP, guanosine monophosphate; IP_3_:R Inositol 1,4,5-triphosphate receptor; SR, sarcoplasmic reticulum; SERCA, Sarco/endoplasmic reticulum Ca^2+^ ATPase; MLCK, myosin light chain kinase; NCX, Na^+^/Ca^2+^ exchanger; PMCA, Plasma membrane Ca^2+^-ATPase.

Relaxation of the corpus cavernosum causes penile erection in response to cholinergic neurotransmission, with nitric oxide (NO) being the most important neurotransmitter. In addition, non-adrenergic non-cholinergic neurotransmission (NANC) transmitters are also found adrenergic nerves (Andersson, 2011; Hannigan, 2016). Further, other mediators are also responsible for modulating cavernous smooth muscle relaxation, such as prostacyclin (PGI_2_) and type 1 and 2 prostaglandins (PGE_1_ and PGE_2_). These prostanoids act on the G_s_ protein-coupled IP, EP_2_ and EP_4_ receptors, culminating in activation of _c_GMP and _c_AMP-dependent protein kinases (PKG and PKA, respectively) which, when activated, phosphorylate various targets such as potassium channels, activating them, and voltage-dependent calcium channels, inhibiting them (**Figure 1**) (Porst, 1996; Angulo et al., 2002; Andersson, 2011).

## Diagnosis of Erectile Dysfunction

Diagnosis of ED is complex because it results from personal, interpersonal and social implications related to disease, involving the identification of the main signs and symptoms presented by the patient and determination of the stage of disease and use of medicines, with the aim of identifying its primary etiology, reduce associated risk factors, and prevent the harmful effects of diseases correlated with dysfunction (Corona et al., 2006; Glina et al., 2014).

Given this limitation, questionnaires that help determine the real development and severity of ED were standardized and validated. Among them, the most commonly used in clinical practice are the Male Sexual Health Inventory (SHIM), which presents five specific questions about ED and the International Index of Erectile Function (IIFE), which has 15 questions related to all phases of male sexual response (Rosen et al., 2002; Ghanem et al., 2012; Rosen and Spiegelman, 2014).

In addition to a complete survey of the patient**’**s sexual, surgical, psychosocial history, and medication use, the diagnosis of ED requires adequate physical examination, as well as an assessment of blood pressure and weight, given the association of the disease with hypertension and obesity. Another crucial point for the diagnosis of this dysfunction is the evaluation and determination of testosterone levels, since low concentrations of this hormone have been related and contributed to the development of ED (Cordero et al., 2010; El Taieb et al., 2019; Irwin, 2019).

Local penile evaluation is another important alternative because it provides information on the presence of penile deformities, elasticity, urethral meatus, testicular consistency, and fibrosis plaques, which are related to penile erection impairment (Shamloul and Ghanem, 2013). Based on the aforementioned assessments, it is possible to differentiate the etiology of disease from psychogenic or organic, and more precisely the target the treatment of ED.

## Treatment of Erectile Dysfunction

Treatment of ED is performed according to the clinical evaluation of the patient, and it can be divided into non-pharmacological and pharmacological. The non-pharmacological therapy is based on lifestyle modifications, including control of glycemic levels and lipid profile (cholesterol and triglycerides), reduction in addition to stopping smoking and alcohol use, as well as the practice of physical activity (Kupelian et al., 2007; Maiorino et al., 2015). There are currently surgical interventions, devices penile devices, and psychotherapy, used as alternatives for non-pharmacological treatment. In addition, there is hormone replacement and the use of drugs that constitute the pharmacological treatment of this disease (Eardley et al., 2010; Hatzimouratidis et al., 2010).

Oral therapy is the first line treatment for erectile dysfunction and involves the use of PDE5 inhibitors such as sildenafil, which is the prototype of the group, tadalafil, vardenafil and iodenafil. Mechanistically, these drugs facilitate erection by inhibiting the enzyme PDE5, which is responsible for cGMP degradation in smooth cavernous muscle. This inhibition results in prolonged cGMP activity, which decreases cytosolic calcium concentrations, maintaining corpus cavernosum relaxation and, thus, promotes penile erection with a success rate of over 65% (Konstantinos and Petros, 2009; Andersson, 2011; Selph and Carson, 2011). However, previous sexual stimulation is essential to increase intracellular NO levels and, consequently, to cGMP generation (Yafi et al., 2016).

Side effects related to PDE5 inhibitor therapy include, mainly, headache, nasal congestion, facial flushing and dyspepsia. The onset of action of the drug is around 30–60 min, lasting approximately 4–8 h. The main contraindications are nitrate-containing compounds, cardiovascular events, non-arteritic ischemic optic neuropathy and α-blockers (Brant et al., 2007; Zelefsky et al., 2014).

Currently, intracavernous and intraurethral therapies include, mainly alprostadil, with a high therapeutic success rate (90%) (Hatzimouratidis and Hatzichristou, 2005; Perimenis et al., 2006), representing the second line of treatment for ED. Its advantages are the rapid onset time, around 10 min, and the quality of penile erections, even in the absence of sexual stimulation (Shamloul and Ghanem, 2013). Additionally, alprostadil is synthetic prostaglandin E_1_, which by binding to EP_2/4_ receptors activates the adenylyl cyclase (AC) signaling pathway, culminating in the increase of cAMP cytoplasmic concentration, which ultimately results in the corpus cavernosum relaxation and, consequently, the penile erection. It has used in intracavernous injection therapy and as a suppository for intrauretal use (Moreland et al., 2003).

Despite the great therapeutic success of the drugs, around 30%–40% of men with ED do not respond to this first line of treatment. Additionally, the use of injectable medications brings priapism as the main risk, which consists of a painful and prolonged penile erection (greater than two hours), regardless of sexual desire and resulting from insufficient penile blood drainage. In this context, refractoriness to conventional treatments contributes to the search for new therapeutic alternatives for ED (Alves et al., 2012; Codevilla et al., 2013; Munk et al., 2019).

## Ion Channels and Erectile Dysfunction

### Large-Conductance, Ca^2+^-Activated K^+^ Channels (BK_Ca_)

The BK_Ca_ channels are highly conductive (150–250 pS) channels, selective for K^+^ (Wu, 2003) with ubiquitous expression on the plasma membranes of all eukaryotic cells. They are activated in a negative feedback mechanism to control plasmatic membrane excitability in response to membrane voltage and increased cytoplasmic Ca^2+^ concentration. Its dysfunction is implicated in several diseases, including erectile dysfunction (Kshatri et al., 2017; Gururaja Rao et al., 2019).

**Figure 2:**
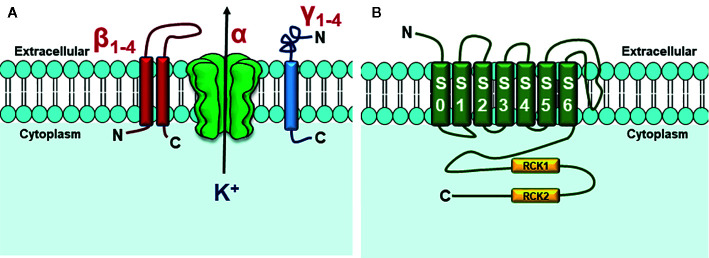
Structure of BK_Ca_ channels. **(A)** BK_Ca_ channels are formed by three protein subunits (α, β, and γ). The α subunit forms the channel pore, permeable to K^+^; **(B)** Seven transmembrane segments form the α subunit. In C-terminal domain, two Ca^2+^-sensitive sites are found, known as regulator of potassium conductance (RCK) 1 and RCK2. Adapted from (Schubert and Nelso, 2001).

These channels are constituted by a tetramer of α subunits, encoded by the Slo gene, which form the channel pore, and auxiliary subunits β_1_–β_4_ and γ_1_–γ_4_ that modulate the physiological activity of these channels (**Figure 2A**) (Contreras et al., 2013; Kshatri et al., 2017). The association with the β_1_ subunit, for example, decreases voltage dependence and increases apparent sensitivity to Ca^2+^ (McManus et al., 1995; Wallner et al., 1995; Lorca et al., 2014a; Lorca et al., 2014b; Balderas et al., 2015).

The α subunits are formed by seven transmembrane segments (S0 – S6) with N-terminal domain located in the extracellular region and C-terminal domain, which has the Ca^2+^ sensor domain, located intracellularly. Voltage sensor comprises segments S0 to S4 and the pore-forming domain includes segments S5 and S6 (**Figure 2B**) (Schubert and Nelso, 2001).

Modulation of BK_Ca_ channels involves several mechanisms. Phosphorylation of channel-forming protein by PKA or PKG may activate or inhibit them, depending on type of smooth muscle evaluated. In pulmonary artery smooth muscle, protein kinase C (PKC) inhibits BK_Ca_ channels, causing pulmonary vasoconstriction (Barman et al., 2004; Werner et al., 2005).

Additionally, Kun and colleagues (Kun et al., 2009) observed that NS11021 (1-(3,5-bis-trifluoromethyl-phenyl)-3-[4-bromo-2-(1h-tetrazol-5-yl)-phenyl]-thiourea), a BKCa opener, increases the currents sensitive to the selective BKCa channel blocker, iberiotoxin (IbTX) in rat isolated corpus cavernosum smooth muscle cells, and reduced [Ca^2+^]i, and tension in penile arteries, leading to relaxation of the intracavernous arteries, being potential targets for the treatment of ED (Kun et al., 2009). The results obtained by Sung and colleagues showed that another activator of these channels, LDD175, improved erectile function in a diabetic rat model. Furthermore, they showed that LDD175 treatment combined with sildenafil had an additive effect on improving the erectile function of diabetic rats (Nilius and Owsianik, 2011; Sung et al., 2017). These findings suggest that BK_Ca_ channels are possible targets for the treatment of ED.

### Small-Conductance, Ca^2+^-Activated K^+^ Channels (SK_Ca_)

**Figure 3:**
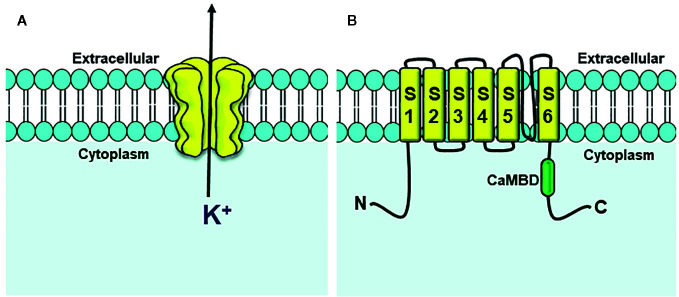
Structure of SK channels. **(A)** Four subunits form the channel pore permeable to K^+^ ion. **(B)** Each channel subunit is formed for six transmembrane segments (S1–S6). In C-terminus, is this located the Calmodulin Binding Domain (CaMBD).

SK are small condutance (10-20 pS) (Kushwah et al., 2018), voltage-independent and cytosolic Ca^2+^ sensivite channels (Cui et al., 2014). The pore of these channels is selective to K^+^ and formed to four subunits (**Figure 3A**) each with six transmembrane α helice domains (S1-S6) and intracellular N- and C-terminus. A loop between the S5 and S6 segments forms the K^+^ selectivity filter (Faber, 2009; Nam et al., 2017) (**Figure 3B**).

Constitutively linked to the channel, in Calmodulin Binding Domain (CaMBD), the calmodulin protein (CaM) mediates the gating of the pore of SK channel (Zhang et al., 2012) after its interaction with Ca^2+^ ions. The rise of cytosolic concentration of Ca^2+^ to about 300–500 nM induces conformation rearrangements in calmodulin and canal subunits, following K^+^ efflux and membrane hyperpolarization (Keen et al., 1999).

These channels are highly conserved in mammals (Adelman et al., 2012), having identified three clones denominated as SK1 (K_Ca_2.1), SK2 (K_Ca_2.2), and SK3 (K_Ca_2.3) encoded by the genes *KCNN1*, *KCNN2*, and *KCNN3*, respectively (Kouba et al., 2020).

SK channels are distributed in various tissues. In particular, a significant abundance of the SK3 channel had been detected in human corpus cavernosum, after analysis of mRNA distribution by PCR-RT. In addition, high SK3-type immunoreactivity have been observed in cavernous and vascular smooth muscle, and in human vascular endothelium (Chen et al., 2004). Comerma-Steffensen and colleagues (2017) observed that SK3 channels were, among the subtypes of SK channels, the most expressed in the corpus cavernosum of mice (Comerma-Steffensen S. et al., 2017).

The expression of these channels in vascular endothelial cells is involved in NO production. Sheng and Braun (Sheng and Braun, 2007) observed that blocking SK channels by apamine, inhibited NO synthesis in human umbilical vein endothelial cells (HUVERs) (Sheng and Braun, 2007). As reviewed by Félétou (Félétou, 2009), events such as the activation of G protein-coupled receptors or shear stress in endothelial cells, induce an increase in the cytosolic concentration of Ca^2+^, activating SK_Ca_ following hyperpolarization of the endothelial cells. As a result, the additional influx of Ca^2+^, favored by increasing electrochemical driving force, and the consequent activation of NO synthase, induces the release of NO by endothelial cells and relaxation of the vascular smooth muscle cells (Félétou, 2009).

In diabetic Sprague-Dawley rats, Zhu and colleagues (Zhu et al., 2010) observed reduction in the frequency of penile erections, after administration of apomorphine, and mRNA and SK3 protein levels reduction in the cavernous tissue of these animals, compared to group of non-diabetic rats (Zhu et al., 2010).

The use of a non-selective activator of K_Ca_2 and K_Ca_3.1 channels (NS309), induced relaxation of the corpus cavernosum of mice in concentration dependent manner. It has also been observed, in transgenic animals with overexpression of SK3 channels, a significant reduction in blood mean pressure, when compared to downregulation and wild SK3 animals. In addition, stimulation of the cavernous nerve improved the erectile function of animals with SK3 overexpression, while this effect was reduced in SK3 downregulation animals (Comerma-Steffensen S. et al., 2017). Furthermore, the relaxation of strips of the corpus cavernosum of mice, induced by NS309, was significantly reduced by the removal of endothelial cells, the use of NO synthase blockers and the use of apamine, which reflects the influence of these channels on endothelial and erectile functions (Comerma-Steffensen S. G. et al., 2017).

Thus, evidence of the participation of SK channels in penile erection, suggests that the use of activators of these channels may be of therapeutic interest for the treatment of ED.

### KCNQ-Encoded Voltage-Dependent K^+^ Channels (K_V_7)

**Figure 4:**
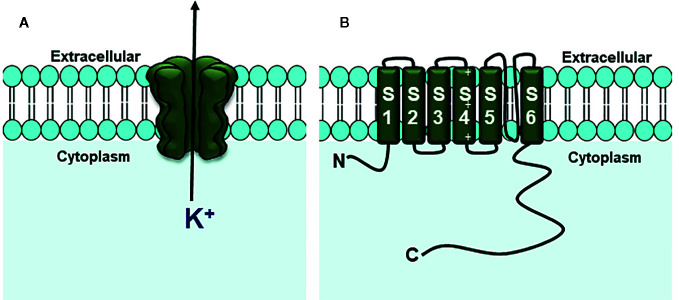
Structure of K_V_7 channels. **(A)** Four subunits form the channel pore permeable to K^+^ ion. **(B)** Each channel subunit is formed for six transmembrane segments (S1–S6).

The voltage-dependent potassium channels encoded by KCNQ (K_v_7) include a family of five members (K_v_7.1 to 7.5 or KCNQ1-5) form subunits of the low-threshold voltage-gated K^+^ channel originally termed the **‘**M-channel**’**, being formed by six transmembrane domains, a single P loop found between S5 and S6, which forms the pore selectivity filter, a fourth positively charged transmembrane domain (S4) that acts as a voltage sensor and a long carboxy terminal tail intracellular (Jentsch, 2000; Brown et al., 2009; Jepps et al., 2013; Lee et al., 2018) (**Figure 4**).

These channels are predominantly expressed in the heart, central nervous system, auditory system and smooth muscle of the vessels, functioning as essential regulators of membrane excitability, playing important physiological roles such as potassium homeostasis, cardiac action potential and neuronal excitability, as well as dysfunctions of the K_v_7 channels are associated with human diseases, including cardiac arrhythmias, epilepsy, deafness, hypertension, and erectile dysfunction (Robbins, 2001; Abbott, 2014; Haick and Byron, 2016).

Additionally, it has been reported the importance of K_v_7 channels in the regulation of vascular and non-vascular smooth muscle tone, and that the KCNQ genes (K_v_7.1, 7.4 and 7.5) are the most expressed subtypes in these muscles, opening a new field of possibilities for pharmacological targeting for the various pathophysiological disorders of the underlying vascular smooth muscle (Greenwood and Ohya, 2009; Soldovieri et al., 2011; Stott et al., 2014).

The pharmacological modulation of these channels in the vessels is responsible for profound changes in the smooth muscle membrane potential and, consequently, in the vasoconstrictor or vasodilator responses of the vascular tone (Jepps et al., 2013). In addition, K_v_7 are also functional endpoints of G_s_-linked receptor agonists (Chadha et al., 2012; Khanamiri et al., 2013; Chadha et al., 2014; Stott et al., 2015).

Several studies have been shown that non-selective blocking of K_v_7.1-7.5 channels by linopirdine and XE991, promote membrane depolarization and concomitant vasoconstriction, leading to an increase in calcium influx through voltage-gated calcium channels (Ca_V_) and consequently inhibit vascular relaxing responses in humans and rodents (Yeung et al., 2007; Yeung et al., 2008; Mackie et al., 2008; Zhong et al., 2010; Jepps et al., 2011; Stott et al., 2015; Lee et al., 2020), which may produce spontaneous contractions in some vessels (Yeung et al., 2007; Mackie et al., 2008; Lee et al., 2020). It has been shown in penile physiology that blocking these channels also impairs arterial relaxation produced by the atrial natriuretic peptide and sodium nitroprusside (SNP), decreasing the cellular concentration of cGMP, essential for the penile erection process (Stott et al., 2015; Jepps et al., 2016).

However, K_v_7 activators (retigabine, ML213 and S-1), hyperpolarize the membrane potential and cause relaxation of pre-contracted vessels, decreasing the Ca^2+^ influx by Ca_V_, resulting in the relaxation of human and rodent arteries (Yeung et al., 2007; Yeung et al., 2008; Mackie et al., 2008; Joshi et al., 2009; Zhong et al., 2010; Chadha et al., 2012). In addition, genes for KCNQ3-5 had been detected in penile arteries, veins and corpus cavernosum, while KCNQ1 was found only in the corpus cavernosum of rats. The activators K_v_7.2-7.5, ML213, and BMS204352, relaxed pre-contracted penile arteries and corpus cavernosum, regardless of nitric oxide synthase or hyperpolarization derived from the endothelium. In contrast, the relaxation promoted by sildenafil and sodium nitroprusside had been reduced by blocking these channels with linopirdine in the penile arteries and the corpus cavernosum (JEPPS et al., 2016).

Therefore, suggesting that K_v_7 channels play an important functional role in all smooth muscle systems, specifically in erectile function, confirming the potential of these channels as new therapeutic targets for erectile dysfunction.

### Transient Receptor Potential Channels (TRP)

TRP channels are a superfamily of ion channels, mostly non-selective for mono and divalent cations, expressed in almost all cell types, in both excitable and non-excitable tissues and participating in various physiological functions such as nociception and muscle contraction (Smani et al., 2015; Moran, 2018).

In mammals, the TRP superfamily is divided into six subfamilies based on their homology sequences and named according to first described member of each subfamily: ankyrin (TRPA), canonical (TRPC), melastatin (TRPM), mucolipine (TRPML), polycystin (TRPP), and vanilloid (TRPV) (Caterina, 2014; Samanta et al., 2018).

**Figure 5:**
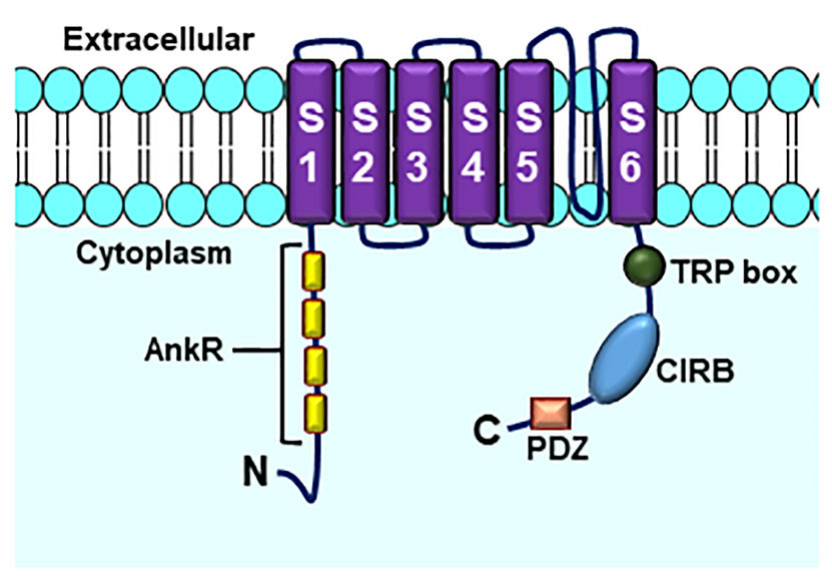
Structure of TRPC channels. AnkR, ankyrin repeats (number differs by subfamily members); TRP box; CIRB, calmodulin- and inositol triphosphate receptor (InsP3R)-binding site; PDZ, amino acid motif binding PDZ domains. Based on (Cao et al., 2018).

Structurally, TRP channels may be homo or heterotetramers, with each channel-forming subunit composed by six transmembrane segments (S1–S6), with the channel pore located between segments S5 and S6 and amino and carboxiterminal domains located intracellularly (**Figure 5**) (Smani et al., 2015; Reggio et al., 2018; Blair et al., 2019).

Activity of these channels can be regulated by a wide variety of stimuli including temperature changes, mechanical forces, lipid mediators such as arachidonic acid (AA) and its metabolites (Del Rocío Cantero et al., 2015) and action of protein kinases such as PKA (Jung et al., 2010).

Recently, studies using negative domain gene transfer to TRP channels have helped to understand the involvement between ED and dysfunctions in TRP channels. TRPC6^DN^ gene transfer reduced cytoplasmic Ca^2+^ concentration in human cavernous smooth muscle and restored erectile function in diabetic rats (Sung et al., 2014). Sung and colleagues (Falzone et al., 2018) showed increased expression of TRPC4 in smooth corpus cavernosum muscle cells of diabetic rats and demonstrated that after TRPC4^DN^ gene transfer, erectile function of diabetic animals was restored (Falzone et al., 2018).

Taken together, these results indicate the possible involvement of TRP channels in pathophysiology of ED, making them potential targets for the development of drugs to treat this pathological condition.

### Ca^2+^-Activated Cl^-^ Channels (CaCC)

**Figure 6:**
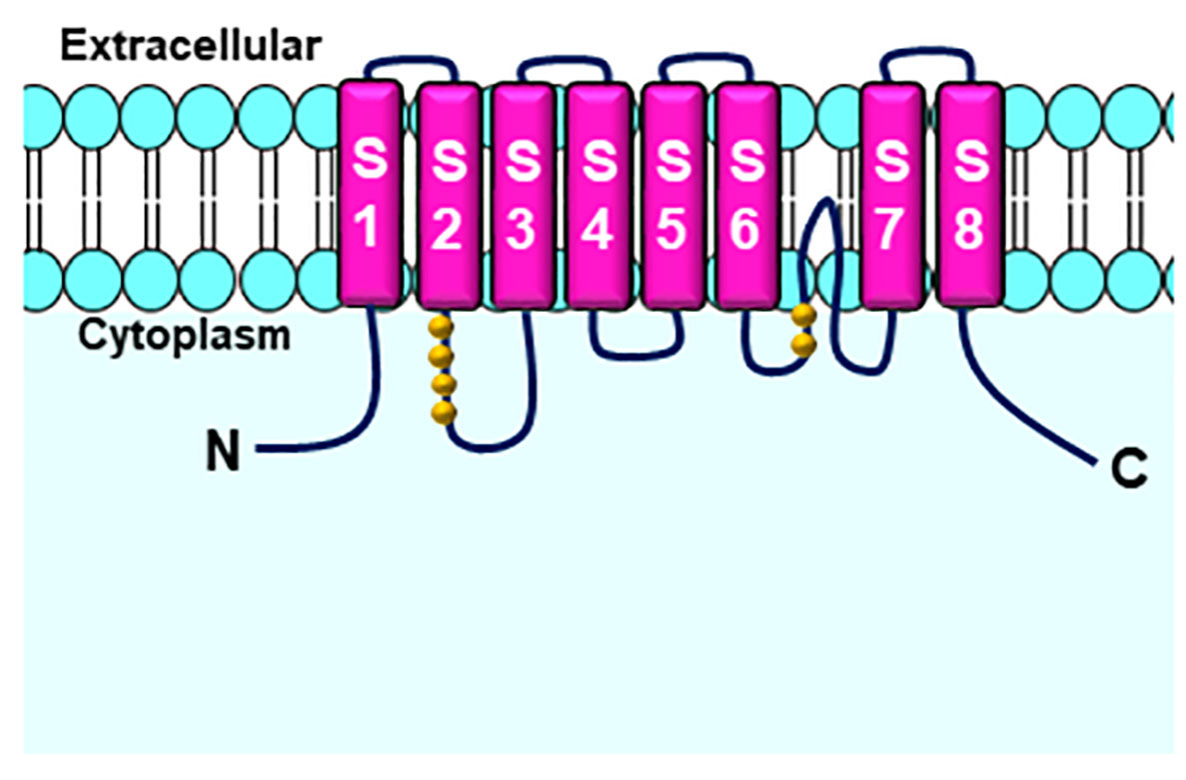
Six transmembrane domains form the Calcium-activated Chloride channels (CaCC)s. Glutamate residues (yellow beads) are potential Ca^2+^ binding sites. Adapted from (Picollo et al., 2015).

CaCC channels belong to a family of transmembrane proteins known as TMEM16 (transmembrane protein with unknown function 16A) (Falzone et al., 2018) (**Figure 6**). Activation of these channels requires an increase in cytoplasmic calcium concentration in the range of 100 nM to 1–2 μM, which may be due to inflow or release from intracellular stocks, allowing Cl^-^ to flow through the plasma membrane (Hartzell et al., 2005; Whorton, 2014; Kamaleddin, 2018).

In smooth muscle, the activation of these channels and consequent chloride efflux induce cell membrane depolarization and voltage-dependent calcium channel activation, resulting in additional Ca^2+^ influx and muscle contraction, having, in particular, an important physiological role in contraction of smooth muscles of the corpus cavernosum, a necessary action for penile flaccidity. Thus, inactivation of these channels by pharmacological agents is a therapeutic alternative for the treatment of ED (Chu and Adaikan, 2008; Linton et al., 2012; Whorton, 2014).

The evidence of Ca^2+^ activated chloride currents in human and rat cavernous smooth muscle cells was demonstrated by Karkanis and colleagues (Karkanis et al., 2003). In this study, the use of 4,4-dithitrostylbene-2,2-disulfonic acid (DNDS) and 4-acetamido-4-isothiocyanostylbene-2,2-disulfonic acid, CaCC blockers, transiently increased intracavernous pressure and prolonged time of erection after cavernous nerve stimulation (Karkanis et al., 2003).

CaCC is associated with the maintenance of basal tone and spontaneous contractions of the corpus cavernosum. The use of two potential erectogenic agents, niflumic acid (NFA) and anthracene-9-carboxylic acid (A9C), CaCC channel blockers, significantly reversed intrinsic contractile activity of the rabbit**’**s corpus cavernosum, as well as the tone of this tissue, after contraction with phenylephrine, histamine or endothelin-1, in a concentration-dependent manner (Linton et al., 2012).

In addition, when using isolated corpus cavernosum from diabetic rabbits, Chu and Adaikan (Leblanc et al., 2015) showed that NFA and A9C were able to increase the nitrergic relaxation of corpus cavernosum smooth muscle of diseased animals, suggesting that inhibition of CaCC may be a viable alternative to diabetes-related erectile dysfunction (Leblanc et al., 2015).

In recent study, by Hannigan and colleagues (Hannigan et al., 2017), the use of two new blockers (T16A_inh_-A01 and CaCC_inh_-A01) was effective in inhibiting CaCC channels, reducing the phenylephrine-induced tone, reinforcing their important role in favor of maintaining penile flaccidity (Hannigan et al., 2017).

## Future Perspectives

**Figure 7:**
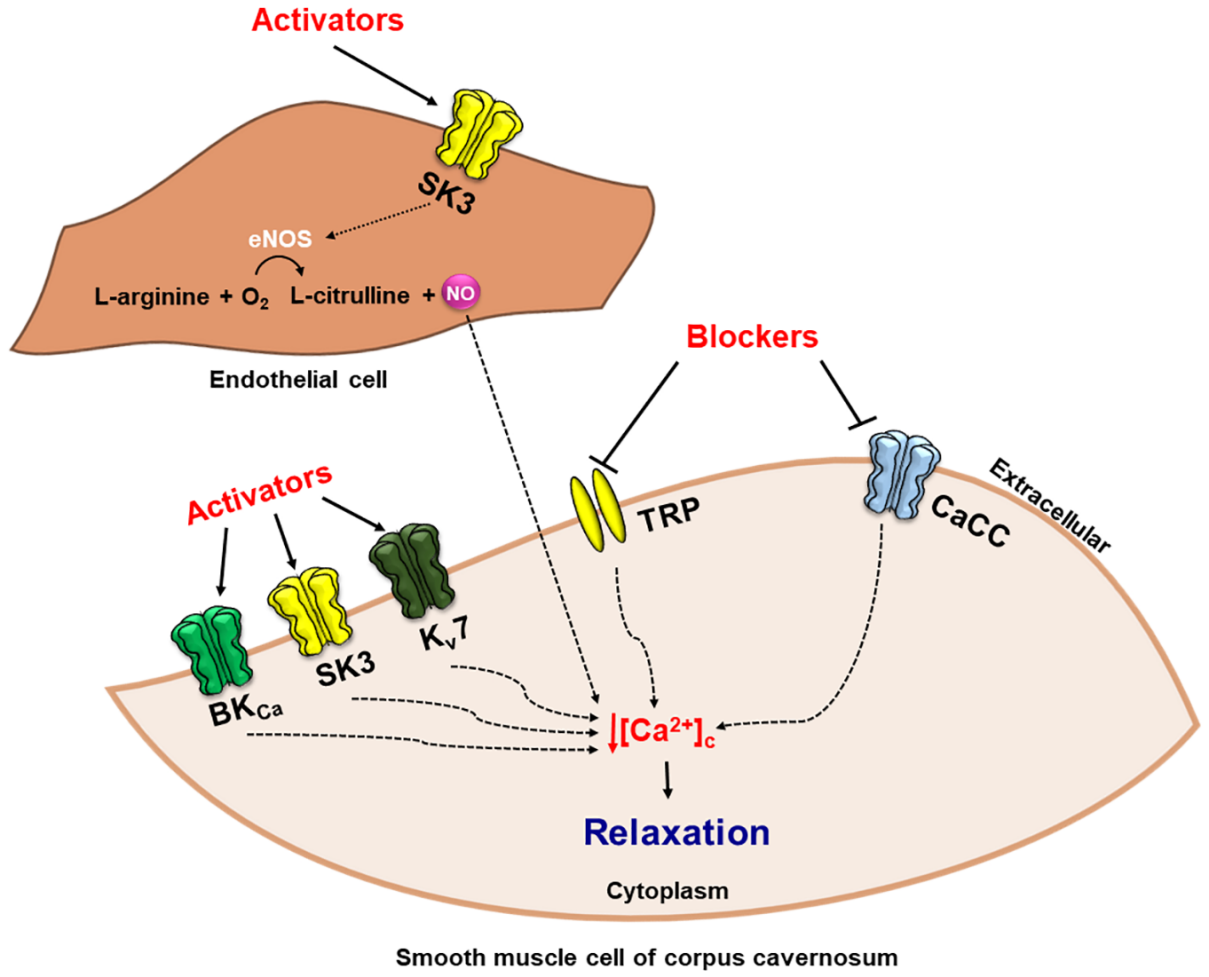
Use of BK_Ca_, SK3, and K_V_7 channels activators and/or transient receptor potential (TRP) and Calcium-activated Chloride channels (CaCC) blockers, induce reduction of cytoplasmic concentration of Ca^2+^, [Ca^2+^]_c_, culminating in relaxation of the cavernous smooth muscle cell and penile erection.

Despite recent advances and researching toward new therapeutic strategies for the treatment of erectile dysfunction, much remains has to be done to clarify the promising role of ion channels in controlling and determining male erectile function, as well as their participation in various other targets of the central and peripheral pathways involved in the regulation of cavernous smooth muscle tone. In this context, searching for new therapeutic targets that favor the penile erection process and the control of other aspects related to sexual function, the use of BK_Ca_, SK3, and K_v_7 channels activators and/or TRPs and CaCC channels inhibitors represent important targets in the development of of possible pro-erectile agents leading to a decrease in cytosolic calcium concentration and consequently relaxation of the cavernous muscle cells, restoring erectile function and favoring penile erection (**Figure 7**). In addition, it is essential that the projections of these new therapeutic agents aim to reduce the side effects promoted by phosphodiesterase inhibitors, which is the most commonly, used first-line therapy for the treatment of ED. However, it is important to recognize that molecular and clinical understanding of sexual function, as well as patient and partner involvement, are critical to the implementation of successful therapy.

Based on the information presented, the modulation of ion channels seems to be a promising alternative for the treatment of erectile dysfunction. Despite this, it is necessary to emphasize the importance of investigating possible adverse effects that can happen after the modulation of ion channels. In this context, possible cardiovascular changes such as cardiac arrhythmias, hypotension or hypertension must have be ruled out to ensure the safe use of these possible new targets.

## Author Contributions

AD, RF, and IS made the major part of research, designed and wrote the manuscript. BS guided the preparation of the work.

## Conflict of Interest

The authors declare that the research was conducted in the absence of any commercial or financial relationships that could be construed as a potential conflict of interest.

## Abbreviations

ED, Erectile dysfunction; BK_Ca_, Large-conductance, Ca^2+^-activated K^+^ channels; TRP, Transient Receptor Potential; CaCC, Calcium-activated Chloride channels; K_V_7, KCNQ-encoded voltage-dependent K^+^ channels; SK_Ca_^2+^, Small-conductance, Ca^2+^-activated K^+^ channels; ROS, Reactive Oxygen Species; eNOS; RhoA, Endothelial nitric oxide synthase; Small G protein GTP-binder; ROCK, Rho-associated protein kinase; MS, Metabolic Syndrome; NO, Nitric Oxide; CNS, Central Nervous System; NA, Norepinephrine; NANC, Non-adrenergic, Non-cholinergic; PGI_2_, Prostacyclin; PGE_1/2_, Prostaglandins E type 1 and 2; CaM, Calmodulin; Ca_V_, Voltage-dependent Ca^2+^ channels; AC, Adenylyl cyclase; PKA, cAMP dependent protein kinase; PKG, cGMP-dependent protein kinase; ATP, Adenosine triphosphate; GTP, Guanosine triphosphate; cAMP, Cyclic adenosine monophosphate; cGMP, Cyclic guanosine monophosphate; IP_3_, Inositol 1,4,5-triphosphate; SR, Sarcoplasmic reticulum; SERCA, Sarco/endoplasmic reticulum Ca^2+^ ATPase; MLCK, Myosin light chain kinase; NCX, Na^+^/Ca^2+^ exchanger; PMCA, Plasma membrane Ca^2+^-ATPase; SHIM, Male Sexual Health Inventory; IIFE, International Index of Erectile Function; PKC, Protein kinase C; RCK_1/2_, Regulator of potassium conductance; TRPA, Transient Receptor Potential Ankyrin; TRPC, Transient Receptor Potential Canonical; TRPM, Transient Receptor Potential Melastatin; TRPML, Transient Receptor Potential Mucolipine; TRPP, Transient Receptor Potential Polycystin; TRPV, Transient Receptor Potential Vanilloid; AA, Arachidonic acid; TMEM16, Transmembrane protein with unknown function 16A; DNDS, 4,4-dithitrostylbene-2,2-disulfonic acid; NFA, Niflumic acid; A9C, Anthracene-9-carboxylic acid.
